# Seminal papers in urology: maintenance Bacillus Calmette-Guerin (BCG) immunotherapy for recurrent Ta, T1 and *carcinoma in situ* transitional cell carcinoma of the bladder: a randomized southwest oncology group study (SWOG-8507)

**DOI:** 10.1186/s12894-023-01352-0

**Published:** 2023-11-24

**Authors:** Jake Tempo, Damien Bolton, Michael O’Callaghan

**Affiliations:** 1 Austin Health, Melbourne, Australia; 2 College of Medicine and Public Health, Flinders University, Adelaide, Australia; 3 Flinders Medical Centre, Urology Unit, Adelaide, Australia; 4 Discipline of Medicine, The University of Adelaide, Adelaide, Australia

**Keywords:** Bladder Cancer, BCG, Randomised Controlled Trial

## Abstract

The South West Oncology Group’s 2000 randomised-control trial investigated the addition of maintenance intravesical bacillus Calmette-Guerin (BCG) to non-muscle invasive urothelial carcinoma (NMIUC) treatment. The results were published when the efficacy of BCG immunotherapy maintenance was unclear.

Randomisation produced two arms, each containing 192 patients assessed to be at high risk of recurrence following induction BCG therapy for NMIUC. The treatment arm went on to receive three successive weekly intravesical and percutaneous BCG administrations at three months, six months and then six monthly for three years from the start of induction therapy.

Recurrence free-survival (RFS), was higher in the maintenance arm with 41% (95%CI 35–49) RFS at five years in the control arm and 60% RFS (53–67 95% CI) in the maintenance arm (*p* < 0.0001). Only 16% of patients in the treatment arm received all of the scheduled maintenance courses of BCG.

The study’s seminal results correlate with contemporary systematic review and have guided international guidelines.

## Background

Urothelial carcinoma is the most common malignancy of the bladder and is typically diagnosed and resected during cystoscopy. Whilst the gold-standard treatment for muscle invasive bladder cancer remains cystectomy, guidelines recommend induction and maintenance courses of intravesical Bacillus Calmette-Guerin (BCG) for intermediate and high-risk Non-Muscle Invasive Bladder Cancer (NMIBC) [1]. The randomised control study conducted by the Southwest Oncology Group discussed in this review is a seminal paper in urology as it was the first to study a repeating three-weekly maintenance regime which resulted in a recurrence-free survival benefit.

The mechanism of BCG on urothelial carcinoma cells begins with the infection of the urothelium resulting in the internalisation of BCG within cancer cells. These cancer cells induce an immune reaction mediated by granulocytes, T-helper cells, dendritic cells, macrophages and immune molecules [2, 3]. Anti-tumour effects are induced by Th1 cells via CD4 + T-cells and CD8 + T lymphocytes [4]. Th2-cell activation occurs through natural killer cells alongside neutrophil recruitment and macrophage activation which target the BCG infected cancer cells [5].

Studies published in the 1990s compared intravesical BCG to doxorubicin and mitomycin and report longer intervals to first recurrence with the former [6, 7]. The length of an induction course became six weeks due to both the understanding that the delayed hypersensitivity reaction from the immune-mediated BCG reaction required at least three weeks of exposure in addition to BCG vials being supplied in batches of six 120mg vials [8]. The purpose of adding BCG maintenance, once the immune stimulation of the induction course has ended, was to increase the time to recurrence with the assumption that this will increase survival [9]. The first small randomised studies comparing induction to short maintenance courses of single instillations either monthly, three monthly or six monthly (with a six-week course) did not demonstrate a recurrence reduction [10–12].

## Appraisal of study methods

The aim of Lamm’s randomised control study was to assess whether maintenance BCG therapy reduces bladder urothelial carcinoma recurrence after induction BCG for NMIBC.

Inclusion criteria required patients with pTa or pT1 urothelial carcinoma of the bladder diagnosed via complete TURBT within six months prior to enrolment. Only patients deemed to be of increased recurrence risk were included, selected by having at least one recurrent tumour within one year, three or more within the most recent six months and/or *carcinoma *in situ (CIS). Exclusion criteria included muscle invasion, prior bladder radiotherapy, patients planned for concomitant chemo- or radiotherapy and those with prior BCG therapy.

All patients received an induction course of 81mg (10.5 ± 9.7*10^8^ colony-forming units) Connaught BCG suspended in 50.5ml of saline into the bladder. They lay prone for 15 min and retained the suspension for 120 min if tolerated. Percutaneous BCG of 0.5ml was administered with a 28-gauge needle. These steps were repeated weekly for six weeks.

All patients who completed this induction course received purified protein derivative skin testing and were divided by their response (< 5mm or > / = 5mm) and also divided by whether they initially had CIS or not. Randomisation took place assigning patients to a BCG maintenance arm or control arm, the latter not receiving further BCG unless they experienced residual or recurring disease.

The maintenance arm received three successive weekly intravesical and percutaneous BCG administrations at three months, six months and then six monthly for three years from the start of induction therapy.

The primary outcome measure to determine efficacy was recurrence-free survival.

The interval to assess recurrence-free survival was two years as this was the median recurrence free survival time observed in a prior BCG study at their institution. A priori power calculations were performed and the accrual goal was reached.

Six hundred sixty patients were recruited from 81 US institutions between December 1985 and December 1988 and 550 were accepted into the study. Twelve patients were excluded due to incomplete information ascertained prior to randomisation and 154 patients had evidence of disease at the time of randomisation and were therefore excluded. Therefore, 192 eligible patients were randomised into both arms of the study. There was evenly balanced demographics for race, age, *CIS,* and skin prick test in each arm.

## Summary of outcomes

The primary aim of the study, determining recurrence free-survival (RFS), was better in the maintenance arm with 41% (95%CI 35–49) RFS at five years in the control arm and 60% RFS (53–67 95% CI) in the maintenance arm (*p* < 0.0001). Maintenance BCG results in disease-free survival of 77 months versus 36 months in the control arm. Maintenance BCG was reported to reduce long-term recurrence compared to induction BCG alone (Five year recurrence free survival 60% (95% CI 53–67%) vs 41% (95% CI 35–49%). Worsening-free survival (WFS) was improved by maintenance BCG with 70% (63–76, 95%CI) WFS at five years in the control arm and 76% (70–83, 95% CI) WFS in the maintenance arm (*p* < 0.04). Five-year survival was 78% (72–84, 95% CI) in the control arm compared to 83% (95% CI 77–88) in maintenance arm however not statistically significant (*p* = 0.08).

*Carcinoma *in situ response was not the primary aim of the study, with the design excluding patients with evidence of disease at three months post induction therapy. Only 50.7% of CIS patients had no evidence of disease at this stage. When these cases were included, along with those who had recurrence at the three-month post-induction checkpoint, 278 cases of CIS were given induction BCG and randomised (117 to the maintenance arm and 116 to the control arm). No significant difference was reported in recurrence rate (56.9% in control arm 54.7% in maintenance arm (*p* = 0.8)) at three months. When all CIS patients were included in the analysis, including those with recurrence after induction therapy, there was an overall response rate of 83.8% in those in the maintenance arm, including patients receiving additional therapies (*p* = 0.004). The conclusion being that for CIS non-responsive to induction BCG, there is benefit of receiving additional BCG regimens.

Despite there being no toxicity events greater than grade 3 in the maintenance arm, tolerance to full maintenance courses was low due to withholding treatment with severe dysuria, fever or malaise. Only 16% of the 243 maintenance cases received all 27 planned BCG instillations over the three years.

## Assessment of evidence

The study performs well under the Cochrane risk of bias assessment. The data end points are quantitative and given the study closed to registrations more than 10 years after recruitment ceased, it provides excellent long-term follow-up of its participants.

The Risk of Bias 2 (RoB2) algorithm reports low risk of bias relating to the following domains; randomisation process, missing outcome data, measurement of the outcome and selection of the reported result [13]. The RoB2 algorithm determined ‘some concerns’ of bias for the domain regarding deviations from intended interventions due to low overall adherence to maintenance regimes and therefore ‘some concerns’ of bias overall. The ‘Intention To Treat’ (ITT) methodology of this study is important when implementing the findings into clinical practice. The ITT methodology preserves randomisation and therefore lowers bias compared to ‘per protocol’ trials and, given adherence to maintenance BCG courses is poor in practice, this is important [14].

Data regarding quality of initial resection and the period length between induction and maintenance start time is omitted and would provide insight into the importance of adherence to regimes.

Percutaneous BCG, given in this study, has been discontinued in clinical practice due to lack of evidence of benefit when given at the time of intravesical treatment [15, 16]. Given that effective immune priming takes time, a contemporary study in which percutaneous BCG was given 21 days prior to intravesical treatment to prime the immune response reported promising results and this protocol warrants further investigation [17].

## Research in context

A systematic review and meta-analysis of ten RCTs (including this one) published in 2018 reported that the addition of maintenance BCG prolongs RFS by 33% (HR 0.67; 95%CI 0.54–0.82; *p* =  < 0.001) and reduces tumour recurrence by 21% (RR 0.79, 95%CI 0.70–0.89; *p* < 0.0001) [18]. The meta-analysis shows a similar effect estimate to the SWOG-8507 trial, with low statistical heterogeneity between trials, increasing our confidence in this result.

Since the global shortage of BCG began with the suspension of BCG Connaught production in 2011 and exacerbated by the COVID-19 pandemic, clinicians must consider rationing BCG. The EORTC 30962 trial demonstrated that reducing the maintenance BCG course length to one year did not impact progression or survival however the disease-free interval was affected when the dose was reduced to a third for one year [19, 20]. The NIMBUS study, reduced overall BCG dose further; to three induction instillations and six maintenance doses throughout one year. The trial was stopped prematurely after the reduced dose regime demonstrated a significantly higher recurrence rate. Despite the Connaught strain of BCG being discontinued, other BCG strains appear to be as effective as determined by meta-analysis [21].

## Conclusion

This study reported improvement in recurrent free-survival and disease-free survival with the addition of maintenance BCG courses at three months, six months and then six monthly for three years from the start of induction therapy. The dissemination of these findings has formed the basis for international guidelines for BCG maintenance regimes.

## Data Availability

This paper uses no primary data.
